# Renal osteodystrophy in Egyptian CKD patients: observations from clinically indicated bone biopsies

**DOI:** 10.1186/s12882-026-04776-6

**Published:** 2026-02-20

**Authors:** Nehal Elshabrawy, Mahmoud M. Sobh, Rasha T. Shemies, Mohamed M. Abdalbary, Ahmed Almenshawy, Hanaa I. Okda, Basma O. Sultan, Ehab E. Eltoraby, Amr El-Husseini

**Affiliations:** 1 Mansoura Nephrology and Dialysis Unit (MNDU), Internal Medicine Department, Mansoura University, Mansoura, 35516 Egypt; 2 Clinical Pathology Department, Faculty of Medicine, Mansoura University, Mansoura, 35516 Egypt; 3 Internal Medicine and Nephrology, Faculty of Medicine, Tanta University, Tanta, 31511 Egypt; 4 Department of Internal Medicine, Nephrology Unit, Faculty of Medicine, Suez Canal University, Ismailia, 41511 Egypt; 5 Mansoura Rheumatology and Immunology Unit, Internal Medicine Department, Mansoura University, Mansoura, 35516 Egypt; 6 Internal Medicine and Nephrology, Division of Nephrology & Bone and Mineral Metabolism, University of Kentucky, Lexington, KY 40536-0298 USA

**Keywords:** Renal osteodystrophy, CKD-MBD, Osteoporosis, Metabolic bone disease, Aluminum intoxication, Adynamic bone disease.

## Abstract

**Background:**

Renal osteodystrophy (ROD) varies among populations. Different genetic and environmental factors and prescription patterns may explain this variation. Egyptian ROD is not sufficiently studied. Although bone biopsy is the gold standard tool, no single study reported the actual ROD spectrum based on bone biopsy in Egypt or Africa. International guidelines must consider the different patterns in developing countries.

**Methodology:**

The ISN-sistership program enabled us to create an Egyptian bone biopsy consortium that provided a nationwide specialized CKD-MBD service. We included all CKD patients who were referred for unexplained bone pain, osteoporosis, or abnormal CKD-MBD laboratory parameters. Bone biopsy was offered for those who had clinical indications.

**Results:**

Over 2 years, 270 patients were recruited: 118 pre-dialysis, 97 on HD, 21 on PD, and 34 excluded. Non-invasive evaluation suggested that high bone turnover prevailed. Fourteen patients consented to the bone biopsy; all were on HD. Unexpectedly, various degrees of positive aluminum staining were present in 93% of biopsied patients, despite negative results in the dialysate water and nonuse of aluminum-based phosphate binders. Root cause analysis was done, triggering an environmental alarm for potential sources in food, water, and drug manufacturing. Aluminum-induced suppression of bone cells was confirmed by low turnover biomarkers in patients with significant aluminum accumulation. Moreover, FGF23 was significantly higher in the same group (z=-2.082, p-value = 0.037).

**Conclusion:**

To conclude, Aluminum bone disease is still on the differential diagnosis list of ROD. Of the small biopsied patients, 93% had variable degrees of positive aluminum staining, and 57% had significant aluminum accumulation. Extra efforts are needed to eliminate this bone toxin.

**Supplementary Information:**

The online version contains supplementary material available at 10.1186/s12882-026-04776-6.

## Background

ROD is defined as bone morphological alterations in patients with chronic kidney disease (CKD). It resembles the skeletal component of chronic kidney disease - mineral and bone disorder (CKD-MBD) and shares in the pathogenesis of cardiovascular diseases, which is the most common cause of death in CKD [1]. These metabolic disturbances increase the fracture risk 4-fold compared to the general population [2, 3].

Major histological types of ROD include high turnover bone diseases (HTBD) as osteitis fibrosa, and mixed uremic osteodystrophy, or low turnover bone diseases (LTBD) as adynamic bone disease (ABD), osteomalacia (OM), and aluminum bone disease. Bone biopsy is the gold standard tool to diagnose ROD. Although it gives a comprehensive idea about bone turnover, mineralization, and volume, bone biopsy is underutilized due to its invasive nature and the lack of experts [4]. Non-invasive tools such as imaging modalities (DEXA: Dual-energy X-ray Absorptiometry, CT: computerized tomography scan, and others) and bone turnover biomarkers (bone-specific alkaline phosphatase (BsAP), tartrate-resistant acid phosphatase 5b (TRAP5b)) were introduced in the management of ROD as a trial to partially replace the bone biopsy [5, 6]. Even though in some patients, the results of these biomarkers cannot discriminate the pattern of bone disease, bone biopsy might be indicated.

Over decades, the prevalence of each pattern of ROD changes among different CKD stages and different races [7]. Historically, HTBD was the commonest pattern, especially in patients on hemodialysis [8]. With the introduction of water treatment systems and the wide use of active vitamin D, aluminum bone disease is disappearing, and the prevalence of ABD is increasing [9, 10]. The pattern of ROD is not the same among different populations. Several ethnic and environmental factors, as well as comorbidities, and varying CKD treatment policies largely contribute to this difference. ABD has been described as the predominant ROD in the United States and Europe, even among patients on dialysis [7]. On the contrary, the Brazilian registry of bone biopsy revealed a predominance of HTBD [11, 12]. A similar trend of higher prevalence of HTBD in HD patients has been reported in some Asian countries [13, 14].

Egypt has more than 7 million CKD patients [15]. This number might be even higher if the lack of an effective ongoing CKD registry is considered. Egyptian ROD is not sufficiently studied; to the best of our knowledge, no single study has reported the actual spectrum of ROD based on bone biopsy in Egyptian patients. Most of the published studies shed light on the biochemical alterations in HD patients, particularly focusing on blood levels of minerals and bone biomarkers. Few studies evaluated the use of imaging in the assessment of ROD. Thus, the spectrum of ROD in Egypt remains poorly investigated; exploration of the current situation to fill this gap of knowledge is still needed. This understanding would influence regional practice guidelines for more suitable population-specific recommendations. As an example, the Japanese Society for Dialysis Therapy sets its own distinct targets and recommendations for serum calcium, phosphorus, and PTH that differ from those outlined by the KDIGO CKD-MBD guidelines [16]. Adhering to these guidelines has led to decreased mortality rates among Japanese patients with CKD [17].

This study aimed to explore the spectrum of ROD among Egyptian patients with CKD. This is thought to improve our understanding of the magnitude of the problem and guide the development of tailored guidelines for CKD-MBD in Egypt.

## Methods

This was a cross-sectional study conducted over two years, starting from the launch of the Egyptian Bone Biopsy Consortium in 2022. The consortium, a not-for-profit organization founded to advance clinical and research bone biopsies for CKD patients, aimed to characterize the full spectrum of ROD in Egypt and establish a national registry for CKD-related bone diseases.

The consortium, which includes nephrologists from across Egypt, initiated a specialized bone biopsy service to enroll CKD patients based on clinical indications. The study was primarily carried out at the Mansoura Nephrology and Dialysis Unit (MNDU), in collaboration with the University of Kentucky as part of the ISN-Sistership Program.

### Patient selection

CKD patients who presented or were referred to our clinics over the past two years were eligible for inclusion if they exhibited unexplained bone pain, fractures, osteoporosis, or abnormal CKD-MBD laboratory parameters. Bone biopsies were offered to patients with clinical indications, such as bone pain or fractures, that could not be explained through non-invasive diagnostic tools.

Patients were excluded from the study if they are younger than 18 years, pregnant, or lactating. Patients with active malignancy, uncontrolled thyrotoxicosis, chronic alcoholism, decompensated chronic liver disease, and life-threatening comorbid conditions such as active infection, systemic illnesses were excluded from the study. Patients who are allergic to tetracycline or not willing to give informed consent were not included in the study. Kidney transplant recipients were not included in our study.

Initial non-invasive assessment of turnover was as follows: low turnover was determined as iPTH < 35 pg/ml in G4&G5 non-dialysis and < 130 pg/ml in G5 dialysis and absence of high alkaline phosphatase. High turnover was determined as iPTH > 585 pg/ml in the absence of low alkaline phosphatase. Unpredictable or average turnover was determined when iPTH values are midway between previous values or where there is a contradiction with alkaline phosphatase. These cut-offs were based on the 2–9 folds of the iPTH rule by KDIGO in dialysis patients. But in the pre-dialysis patients, these numbers were based on the authors’ opinion, as no current consensus in that population. These cut-offs were not validated and were used for descriptive purposes only.

### Material and clinical samples

The following clinical and laboratory data were collected at the time of presentation or just before bone biopsy was taken in the biopsied cases; personal data (Age, sex, residency, smoking and marital state), CKD-related history and medications, and history of fractures or bone pain (assessed by modified bone pain score) as shown in Tables 1 and 2 [18]. All patients underwent basic laboratory investigations, including serum calcium, albumin, phosphorus, and iPTH. 25 (OH) vitamin D levels, alkaline phosphatase, and bone mineral density assessment by DEXA scan were done for selected cases, particularly those with moderate to severe pain. In the biopsied cohort, novel turnover biomarkers, klotho, FGF-23, and sclerostin levels were further included in the work up. Intact parathyroid hormone (iPTH) was measured by cobas e411 (Roche diagnostics) Electro-Chemiluminescence assay: normal range: 15 to 65 pg/ml; intra-assay coefficient of variation: <5%. Serum sclerostin level was measured using (TECO^®^ Sclerostin HS ELISA Kit, LOT no 220822, Switzerland). Quidel kits (San Diego, CA) were used for fibroblast growth factor-23, a-Klotho, bone-specific alkaline phosphatase, and tartrate-resistant acid phosphatase 5b assay. All measurements were performed using enzyme-linked immunosorbent assay. Serum aluminum was assessed by Inductively Coupled Plasma (ICP) in Egypt. Unfortunately, the lab experience was not enough to determine the accurate measurement of serum aluminum, despite that we included the results in our analysis.

Regarding BALP, we have included the assay-specific reference ranges based on the MicroVue BAP EIA, where normal values are 11.6–42.7 U/L for women and 15.0–41.3 U/L for adult men. Cut-offs applied to define high bone turnover were BALP levels above 43 U/L, whereas values approaching the lower limits of these ranges were considered indicative of low turnover. In addition, the lower detection limit of the assay has been reported as 0.7 U/L, ensuring reliable detection of low BALP concentrations with linearity up to 150 U/L. higher concentration can be assessed using dilution. About TRAP5b, we incorporated reference values with mean ± SD levels of 4.3 ± 1.4 U/L in adult men and 4.0 ± 1.5 U/L in women. Values exceeding the upper expected limits for each group were interpreted as evidence of increased osteoclastic activity, while low-range values were considered consistent with reduced resorption. The assay’s analytical sensitivity has also been added, with a minimum detection limit of 0.2 U/L, allowing accurate measurement even at very low TRAP5b levels with linearity up to 15 U/L. Higher concentrations can also be assessed using dilution.

Biopsies were processed and analyzed under the supervision of experts from the Division of Nephrology, Bone and Mineral Metabolism at the University of Kentucky, USA. Anterior iliac crest bone biopsies were performed using a vertical approach. Bone specimens were processed without removal of the mineral and analyzed as described previously [19]. 30% or more of the trabecular bone surface stained with aluminum special stains was used as a cut-off point to define a significant amount of aluminum toxicity [20, 21]. Patients with 30% or more were identified as (AL+) group, and those who had less than 30% were identified as (AL-) group.

### Statistical analysis

The IBM SPSS software (version 25) was used to perform statistical analysis. The normality test of continuous variables was carried out using the Kolmogorov-Smirnov criterion. The Shapiro–Wilk test was used when the number of patients within this variable was less than 50. The categorical data are presented in absolute and relative percentages and frequencies, while continuous data is presented by mean values, with ± standard deviations if they follow a normal distribution and median (interquartile range) if they do not follow the normal curve. The independent samples t-test was used to test the correlation between a quantitative continuous variable that follows a normal curve and a qualitative variable with two categories. Mann-Whitney was used to test the correlation between a quantitative continuous variable that does not follow a normal curve and a qualitative variable with two categories. Correlation tests between two continuous variables were performed using the Pearson correlation coefficient if both variables follow a normal curve. In the case that both continuous variables do not follow the normal curve, correlation tests between two continuous variables were performed using the Spearman correlation coefficient. A p-value of less than 0.05 was considered statistically significant.

## Results

**Fig. 1:**
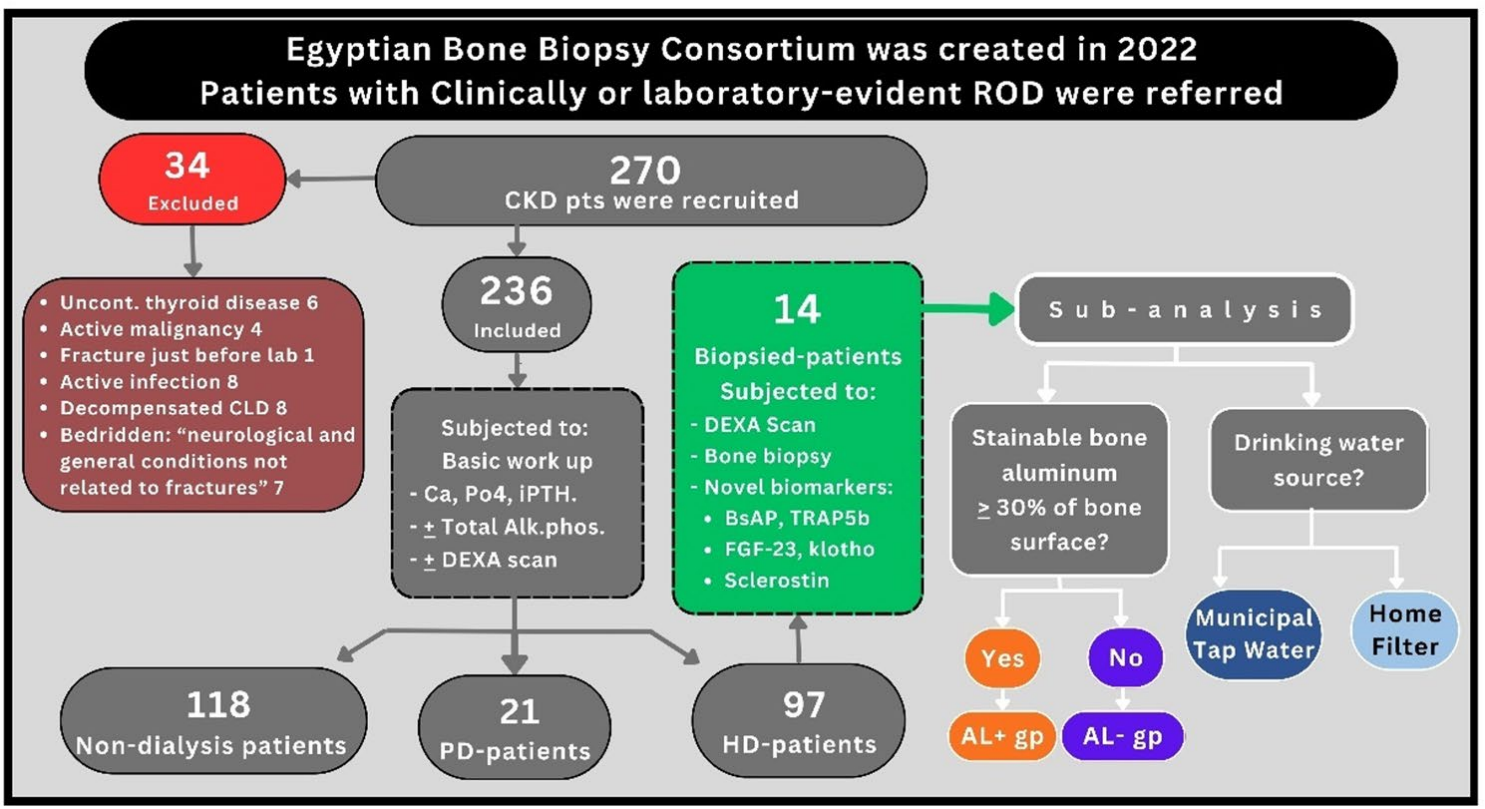
Study flow chart. BsAP: Bone specific alkaline phosphatase, Trap5b: Tartrate resistant acid phosphatase

Our study included patients from different geographic regions in Egypt. Two hundred and seventy patients with CKD were recruited. Figure 1 shows the study flowchart describing the exclusion and classifying the recruited patients.

The general demographic and clinical characteristics of the patients included are depicted in Tables 1 and 2. Only 29 patients had GFR > 30 ml/min. All patients who underwent bone biopsy were in the HD group. Bone pain was present in 43%, 69%, and 19% of patients in the non-dialysis, HD, and PD groups, respectively. Similarly, patients in the HD group showed a higher prevalence of previous fractures (23%) compared to those in the non-dialysis group (3.4%). Of the patients who underwent bone biopsy, 30% had experienced a previous fracture, as shown in Table 2, and all of them had bone pain at the time of presentation. Patients in the HD group showed the lowest calcium (8.7 + 1.3 mg/dl) and the highest iPTH levels (483 – IQR: 563). Based on the non-invasive evaluation of bone turnover using iPTH and alkaline phosphatase, HTBD was predicted in 20%, 29%, and 24% of patients in the non-dialysis, HD, and PD groups, respectively. Whereas the mean iPTH in the biopsied group was 243 + 107 mg/dl, this was dictated by the sampling effect of biopsy indications. Osteoporosis was diagnosed in 43% and 64% of patients who had their bone mineral density evaluated by DEXA scan in the non-dialysis and HD groups, respectively. Only two patients underwent parathyroidectomy; both were maintained on HD.

**Table 1:** Demographics and clinical data

General clinical data	Non-dialysis (118)	HD (97)	PD (21)
Age	58 (19)	54 (24)	48 (19) *
Males	73 (62%)	62 (64%)	11 (53%)
Rural	54 (46%)	43 (44%)	11 (53%)
**Smoking** - Former: - Current	20 (17%) 11 (9%)	14 (14%) 17 (18%)	1 (5%) 2 (10%)
BMI	30 (5)	27 (5) *	28 (5) *
**Comorbidities**: - DM - HTN - IHD - HF - CLD^A^ (Child Pugh score A or B) - Hypothyroidism	46 (39%) 101 (86%) 20 (16%) 6 (5%) 4 (3%) 9 (8%)	13 (13%) 76 (78%) 16 (17%) 11 (11%) 9 (9%) 2 (2%)	5 (24%) 17 (81%) 1 (5%) 1 (5%) 0 (0%) 0 (0%)
**CKD etiology**: - DM - HTN^B^ - GN - Ch. PN / stones - Drug-induced - Others/ Unknown CKD vintage (years) ^C^ HD vintage (years) PD vintage (by months) CKD-Stages / # (%) of patients with residual Kidney function:	36 (31%) 20 (17%) 8 (7%) 9 (7.4%) 15 (13%) 30 (26%) 1.5 (3.5) ---- ---- - G2: 2 (2%) - G3a: 3 (3.5%) - G3b: 24 (20%) - G4: 48 (41%) - G5: 41 (43%)	9 (9%) 25 (26%) 17 (18%) 6 (6%) 14 (14%) 58 (50%) 6 (5) 3 (4) ----- 41 (42%) **UOP**: - No: 104 (77%) - < 0.5 L: 23 (17%) - ≥ 0.5 L: 8 (6%)	3 (14%) 1 (5%) 2 (10%) 3 (10%) 0 12 (57%) 6 (7) 4 (3.3) * 5 (12.5) 16 (76%) **UOP**: - No: 5 (24%) - < 0.5 L: 1 (5%) - ≥ 0.5 L: 15 (71%)
**Bone pain** ^**D**^ - No - Mild - Moderate - Severe	67 (57%) 31(26%) 19 (16%) 1 (0.8%)	30 (31%) 7 (7%) 50 (52%) 10 (10%)	17 (81%) 4 (19%) 0 0
**History of Fracture** -low trauma -High trauma	4 (3.4%) 2 (50%) 2 (50%)	22 (23%) 9 (41%) 13 (59%)	2 (10%) 2 (100%) 0 (0%)

* All continuous variables are expressed as median and IQR except those with*are normally distributed and expressed as mean (standard deviation)

A: Patients with chronic liver disease class C by the Child Pugh classification were excluded from the study; all included patients were either class A or B

B: Hypertension was considered the original kidney disease if there was long-standing hypertension with poor control, malignant hypertension, or hypertensive retinopathy before the onset of CKD

C: Duration since diagnosis of CKD till current referral date

D: **Mild pain**: Pain is noticed but can be ignored with no interference with any activities. **Moderate pain**: Pain cannot be ignored and interferes with physical activity. **Severe pain**: Pain interferes with less than ordinary physical activity and may limit your sleep

**Table 2:** Laboratory parameters and prescription patterns:

	Non-dialysis (118)	HD (97)	PD (21)
HB gm/dl	10.9 (2.2)	11 (1.9)	10.4 (1.5) *
S. Alb gm/dl	3.9 (0.4)	3.9 (0.4) *	3.3 (0.7) *
Cor. Ca mg/dl	9 (0.8)	8.7 (1.3) *	8.9 (0.8) *
Po4 mg/dl	4.4 (1.1)	4.9 (2.5) *	5 (2.9)
iPTH pg/ml	118 (138)	483 (563)	260 (364)
25 (OH) Vit D level ng/mL	31 (21)	21 (10)	17 (9) *
Total Alk. phos. U/L (ref. up to 270)	132 (100) *	367 (479)	--
S. Mg mg/dl	2 (0.4)	2.3 (0.3) *	2.2 (0.6) *
**Apparent turnover state** ^A^:			
- Low - Unpredictable or average - High	7 (6%) 87 (74%) 24 (20%)	24 (25%) 45 (46%) 28 (29%)	4 (19%) 12 (57%) 5 (24%)
**DEXA scan**: - Normal - Osteopenia - Osteoporosis **T-score**: - Spine - Femur neck - Forearm	2 (14%) 5 (36%) 7 (50%) -1.9 (1.4) -0.7 (1.3) -1.8 (1.9)	1 (4%) 9 (32%) 18 (64%) -1.8 (1.5) * -1.8 (1) * -2.7 (1.9) *	0 (0%) 2 (66%) 1 (34%) -1.6 (1.3)* -1.3 (1.6)* -1.3 (1.6)*
**Po4 binder**: - Ca only - Sevelamer only - Sevelamer + Ca	68 (58%) 0 0	71 (73%) 13 (13%) 10 (10%)	14 (67%) 0 3 (14%)
**Type of Ca**: - Ca carbonate - Ca Acetate - Ca Citrate	48 (41%) 19 (16%) 1 (1%)	53 (55%) 27 (28%) 1 (1%)	12 (57%) 4 (19%) 1 (5%)
**Vit D**: - Native only - Alfacalcidol only - Both used:	19 (16%) 26 (22%) 6 (5%)	3 (3%) 36 (37%) 1 (1%)	0 7 (33%) 1 (5%)
**Cinacalcet**	2 (2%)	14 (14%)	2 (10%)
**ESA**	34 (29%)	65 (66%)	17 (81%)
**Doses**: - Elemental Ca (mg/day) - Sevelamer (mg/day) - Native vit D (unit/day) - Alfacalcidol (mcg/day) - Cinacalcet (mg/day) - ESA (unit/month)	420 (111) XXX 2500 (2800) 0.5 (0.25) 60 16,000 (4000)	531 (246) 2300 (1300) * 5000 (6666) 0.5 (0.25) 30 (30) 16,000 (4000)	512 (288) * 3367 (1358) 2222 0.75 (1) 60 16,000 (8000)
**PPI**: - On demand - Daily	12 (10%) 20 (17%)	10 (10%) 41 (42%)	1 (5%) 3 (14%)
**Steroids** ^**B**^ - Only previous exposure: - Currently using:	5 (4%) 2 (2%)	6 (6%) 5 (5%)	0 2 (10%)
**Anti-Osteoporotic**: - Bisphosphonates - Teriparatide	3 (3%) 1 (1%)	1 (1%) 0	0 0
**Oral HCO3 users**:	62 (53%)	5 (5%)	0
**Mg supplementation**	33 (28%)	1 (1%)	4

All continuous variables are expressed as median and IQR except those with*are normally distributed and expressed as mean (standard deviation)

Variables that were not available in most patients (Number of patients subjected to that test in non-dialysis /HD/PD groups): Vit D level (24/26/4), Total Alk Phos (23/29/0), S. Mg (5/75/4), DEXA scan (14/38/3)

**A**: Low turnover was determined as iPTH < 35 pg/ml in G4&G5 non-dialysis and < 130 pg/ml in G5 dialysis and absence of high alkaline phosphatase. High turnover was determined as iPTH > 585 pg/ml in the absence of low alkaline phosphatase. Unpredictable or average turnover was determined when iPTH values are midway between previous values or where there is a contradiction with alkaline phosphatase

**B**: Steroid usage was documented if given a dose equivalent to or higher than 7.5 mg prednisolone for more than 1 mo

### Bone biopsy results

**Fig. 2:**
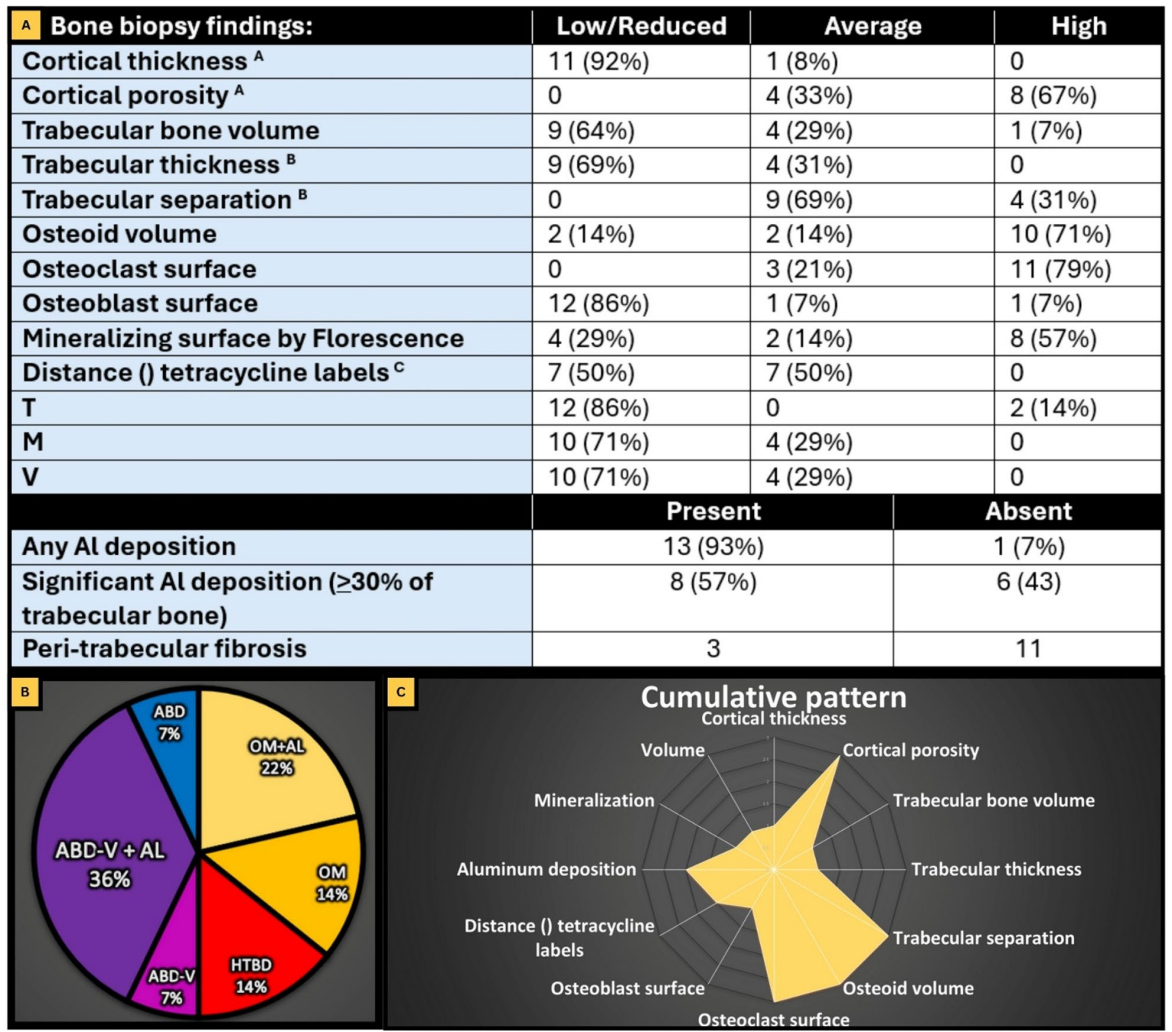
Main biopsy results: Section **A**: Table for different variables in the biopsy reports: **A**: 2 biopsies had no cortical tissue to be examined. **B**: 1 biopsy missed any comment about trabecular thickness. **C**: 5 biopsied had only single label, they were represented in the previous table as “low” as we were sure the patients had received the tetracycline in the appropriate timing. Section **B**: Pie chart for the final diagnosis, where aluminum accumulation was considered significant and added to the final diagnosis only if it reached 30% or more of the stained trabecular bone volume. * Adynamic bone disease – variant (ABD-V) is an ill-described variant of ABD that is characterized by high osteoclast and low osteoblast indices. These relatively higher resorption parameters may reflect a negative bone balance leading to faster loss of bone volume. Section **C**: Radar plot for the cumulative pattern for bone biopsy variables

None of the biopsied patients were maintained on cinacalcet, sevelamer, or aluminum-containing phosphate binders, at least for 6 months before biopsy was taken. Only one of the biopsied patients was on steroids and bisphosphonates, as shown in Table 2. Biopsy findings and final diagnoses were summarized in Fig. 2.

**Fig. 3:**
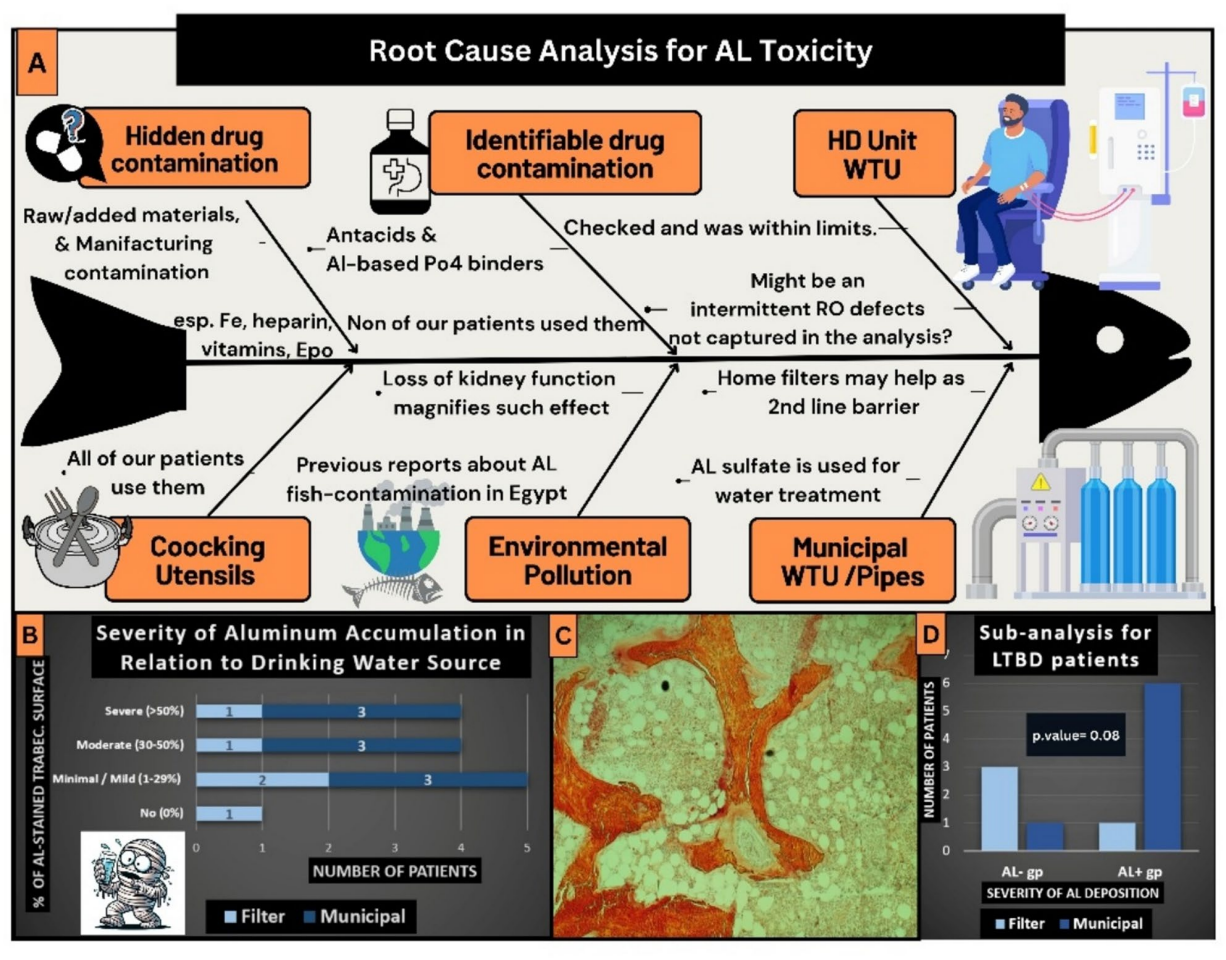
Root course analysis for aluminum toxicity. WTU: water treatment unit, AL: Aluminum, (AL + gp): group of patients with percentage of aluminum-stained bone surface > 30%, the rest of the patients are (AL-gp). Section **A**: In the current study, the level of aluminum was checked in the dialysate and the result was negative. Furthermore, none of our patients have received any aluminum-based phosphate binders. Since aluminum sulfate is used in the municipal water treatment units to purify water, drinking water was on our suspicious list. Subgrouping, as in the study flow chart, helped to guide this analysis; In section **B**, we explored the source of drinking water in each grade of aluminum toxicity in the bone. In section **D**, when analyzing biopsied patients with low turnover states and checking their drinking water sources, 7 patients used municipal tap water and 4 used home filter. The ratios for municipal tap water drinkers to home filter users were 6:1 in the (AL+) group and 1:3 in the (AL-) group, with a near-significant Fisher’s exact test result (p-value = 0.08). During this sub-analysis, one patient was excluded due to marked iron deposition. Any breach in the aluminum removal process in the final steps at central WTUs, or pipes contamination, would directly cause significant aluminum amounts to pass to drinking water. Conventional home water filters might help as a second-line barrier. Environmental pollution is another concern, as mentioned in the discussion section. This represents a speculative suggestion, not true evidence of environmental contamination. Section **C** shows an image from one of our cases with positive aluminum stains where approximately 80% of the trabecular surface exhibits aluminum deposits at the osteoid-bone interface

86% of the biopsied group had low turnover, while only 14% had high turnover. 71% of the patients had abnormal mineralization and reduced bone volume. Adynamic bone disease has been revealed as the most common ROD type in our biopsied group. Surprisingly, various degrees of aluminum staining were identified in 93% of cases. A significant amount of aluminum-stained bone surface (30% or more of trabecular surface at the osteoid-bone interface) was present in 57% of our cases, which was labelled as (AL+) group. Consequently, 86% of the biopsied patients had a low turnover state. Root cause analysis for detecting the possible source of aluminum toxicity was done and summarized in Fig. 3.

**Fig. 4:**
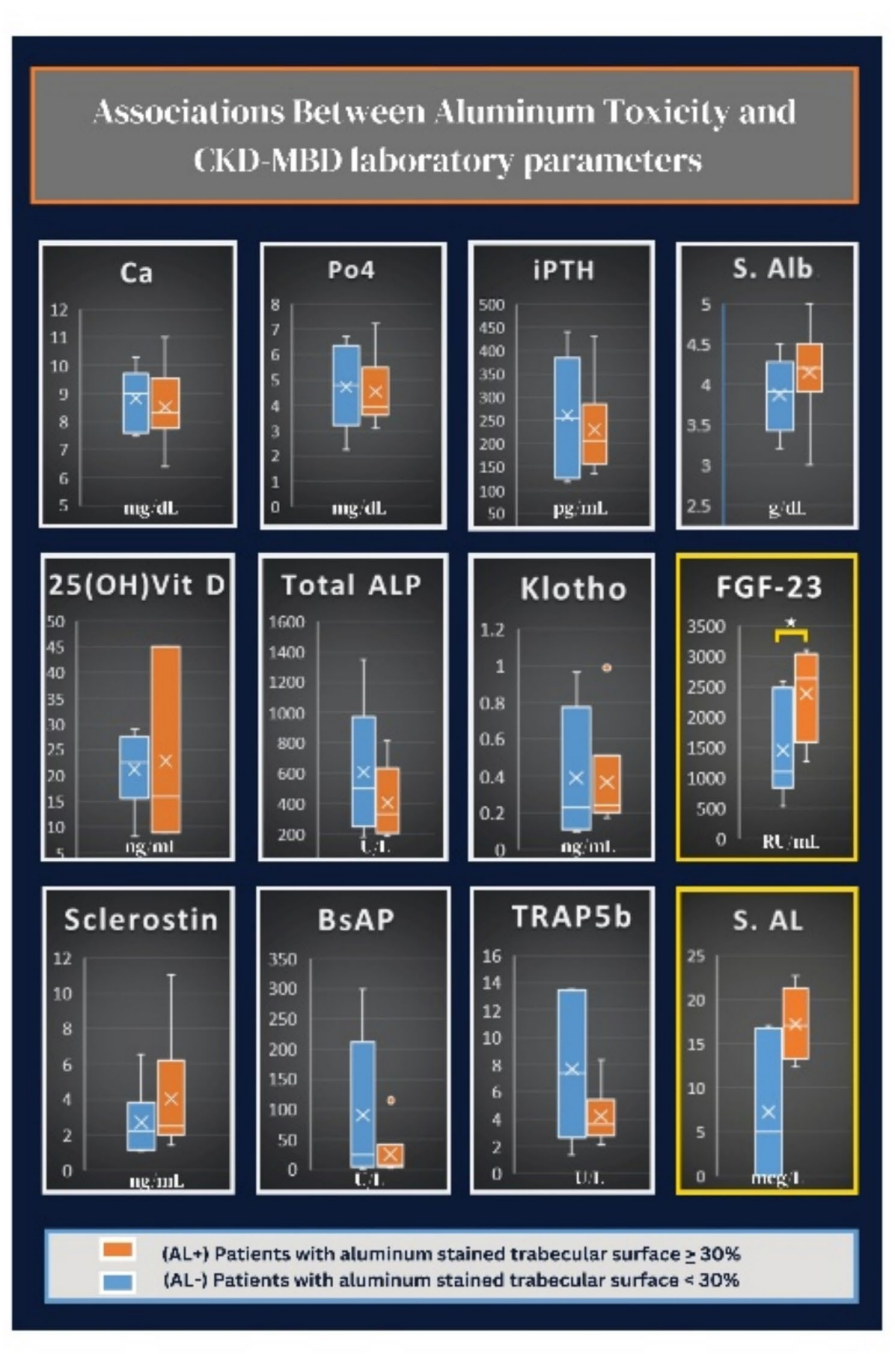
Associations between aluminum toxicity and different CKD-MBD laboratory parameters

The pathobiological effects of aluminum were evident, not only in the bone but also in the sera of patients with significant aluminum deposition “(AL+) group.” Old and novel bone turnover biomarkers showed evidence of suppressed osteoblasts and osteoclasts, as indicated by novel turnover biomarkers in Fig. 4, although it was statistically insignificant due to the small number of biopsied patients. Serum aluminum (S. AL) levels were higher in the AL + group compared to the AL- group (*p* = 0.052). We re-emphasize that our serum aluminum results were not reliable due to the lack of experience in our laboratory for this test.

FGF-23 levels were significantly elevated in the AL + group (*p* = 0.041), with a median of 2626 pg/mL compared to 1096 pg/mL in the AL- group. This finding implies a potential link between aluminum exposure and phosphate metabolism. The exact statistical value for each parameter is shown in Supplementary Table 1.

## Discussion

Health disparities disproportionately affect CKD care all over the world. Genetic and non-genetic factors are thought to contribute to disparities in CKD progression and its consequent/associated morbidities. ROD is particularly an understudied issue among all other known CKD-associated morbidities, considering the known restrictions on performing invasive bone histopathological examination, which in turn limits further understanding of the disease. Unfortunately, the situation is further complicated in the developing world due to limited resources, a lack of experts, and a high socioeconomic burden. The only available data are superficial registries denoting the levels of non-invasive lab values as iPTH [22]. Genetic, environmental, as well as ethnic factors are thought to affect the ROD pattern even before reaching end-stage kidney disease [23]. Little is known about ROD in low-resource settings. Although old Egyptians were pioneers in bone diseases, their descendants are still late behind [24]. No single study has described the histopathologic characteristics of ROD in Egypt or even in the African continent before. Of note, patients living in underprivileged communities are not only diagnosed late but are also likely to have complications at the time of presentation. Lack of awareness, late referrals, and irregular follow-up contribute to this dilemma.

The present study shows that 84% of patients in the non-dialysis group presented at advanced CKD stages (G4 and G5). Among dialysis patients, there was a noticeably low prevalence of DM. This may reflect relatively higher cardiovascular mortality in this sub-population. Increased cardiovascular mortality would be aggravated by the trending prescription patterns that promote a positive calcium balance and vascular calcification. Accordingly, cardiovascular mortality preceded the development of a debilitating bone disease, leading them to have lower odds of being captured in such study. Lower prevalence of bone pain among PD patients can be explained by the relatively higher residual kidney function. Those patients were more likely to present with abnormal laboratory parameters rather than pain or fractures. Of note, higher education and awareness are expected in our PD patients as it is mandatory for the self-care nature of this treatment category. Based on the sampling approach used in this study, patients without evident bone disorders and within target laboratory parameters were less likely to be included in the study.

**Fig. 5:**
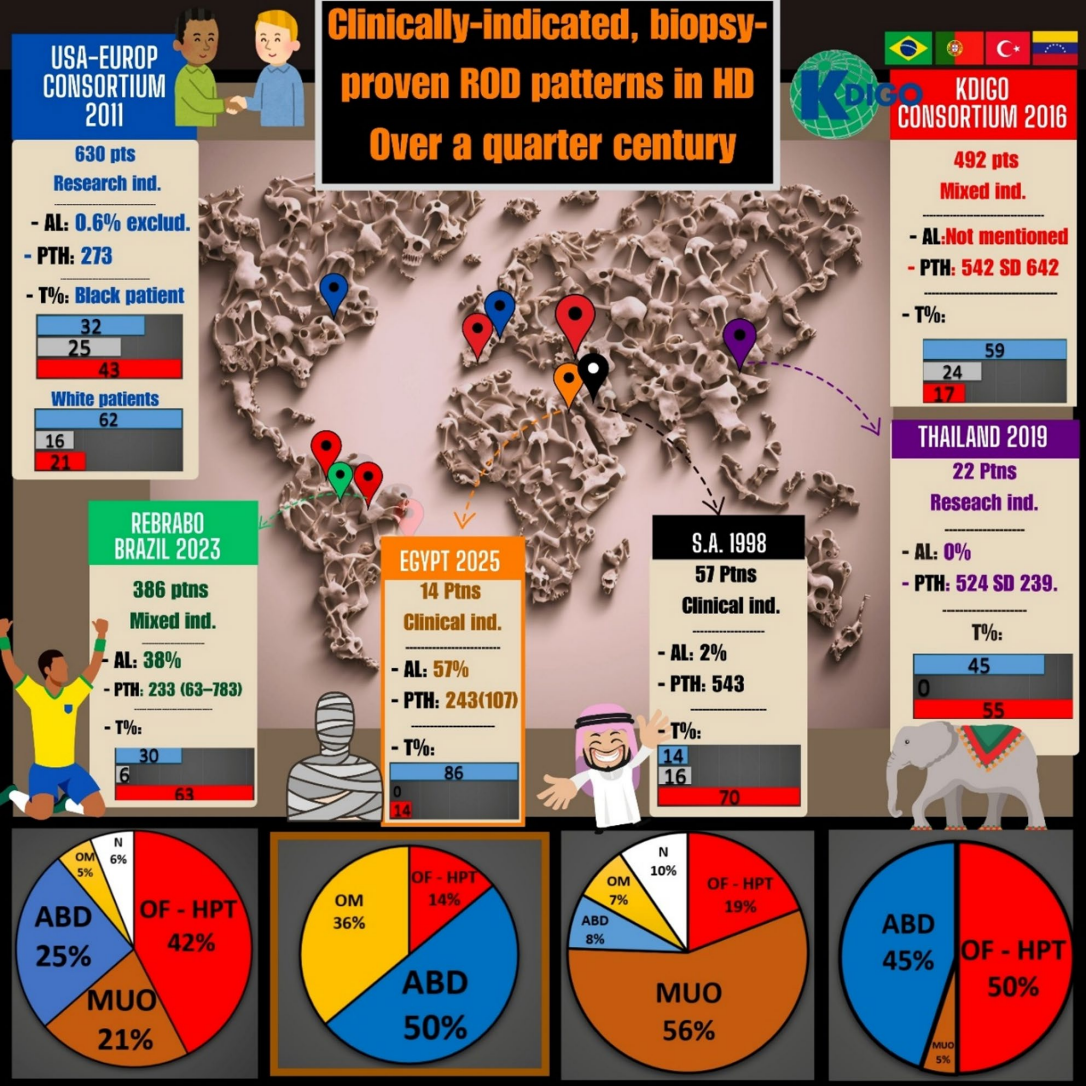
Clinical ROD patterns in the last 1/4 century: Although the USA-European − 2011, and Thailand − 2019 consortia were research-based, we included them, representing the largest number of patients and the latest in Asia, respectively. Both studies highlighted the prevalence of aluminum toxicity in their cohorts. More recent cohorts are present, but we preferred to choose those who mentioned the exact prevalence of aluminum bone disease. T: turnover distribution (blue: low, grey: average, red: high), S.A: Saudi Arabia, REBRABO: The Brazilian Registry of Bone Biopsy, OM: Osteomalacia, ABD: Adynamic bone disease, HPT: hyperparathyroidism, OF: Osteitis fibrosa, MUO: Mixed uremic osteodystrophy

The indications of bone biopsy must be taken into consideration when exploring different cohorts of patients for their ROD patterns. The indication of bone biopsy would impact the prevalence of different ROD types, as it serves as the main inclusion/exclusion criterion. Research indications - where biopsy is taken usually from asymptomatic patients - carry higher odds of normal results. On the other hand, cohorts based on clinical indications – as in our study – would include the worst cases in the grey zone of non-invasive turnover evaluation. Our bone biopsy results were consistent with the ROD pattern shift from high toward low turnover bone disease reported in most cohorts from the USA and Europe, although the cause of low turnover here is obviously different [7, 10]. HTBD had the upper hand in other earlier cohorts [14, 25, 26]. Figure 5 compares our patients to the largest and most recent cohorts, which include dialysis patients biopsied for a clinical indication in the current era. The two main peculiar features of our biopsied group results were aluminum accumulation and ABD variants (ABD-V).

Over the past 30 years, the prevalence of significant biopsy-proven aluminum bone disease has dropped from about 70% to less than 5% [21]. “Aluminum-induced bone disease has virtually disappeared with dialysis water treatment and restricting Al-OH intake in dialysis patients.” This reassuring statement has been repeated in earlier ROD reports and consensuses [27, 28]. Obviously, we have a different situation. In our biopsied group, 93% had various degrees of positive aluminum staining, and 57% had a significant amount of aluminum deposition (> 30% of trabecular surface), labelled as (AL+) group. This staining was not an epiphenomenon, as the pathobiological effects of aluminum on bone were evident, particularly defective mineralization and uncoupling. Defective mineralization was present in ten out of fourteen patients; seven of them had a significant amount of aluminum. Furthermore, Uncoupling – low osteoblast and high osteoblast indices - was present in nine patients, six of them had a significant amount of aluminum. Ongoing bone resorption and suppressed formation create a negative bone balance, which leads to low bone volume. This slowly formed bone is inadequately mineralized, which would further pave the way for low-trauma fractures [21].

This result seems surprising in view of the “supposed” regular surveillance of dialysis water treatment units and the negative history of AL-OH intake by our patients. Therefore, we have re-checked the aluminum levels in random samples from several water treatment units, but the results were negative. The potential source of aluminum was further explored, proposing either an episodic dialysate water contamination that we missed when the recheck was made, or maybe another probable source. Figure 3 summarizes the possible sources of aluminum intoxication in a root cause analysis. Out of these root causes, the following are common among Egyptian HD patients: multivitamins abuse, underutilization of home water filter, environmental pollution, and the use of aluminum utensils [29–33]. Regarding multivitamins, routine prescription of injectable vitamin B complex is one of the trending medical traditions in patients with various chronic diseases, not only HD patients, mostly due to their placebo effect rather than correcting actual deficiencies. Previous reports showed that aluminum contamination may occur during drug manufacturing either in raw materials or during the commercial manufacturing process [30, 31]. Environmental pollution is another concern, particularly in regions near aluminum-related industries where waste might be disposed of in rivers and lakes. Fish consumption in these regions was linked to aluminum toxicity in previous reports from Egypt [32, 33]. This environmental problem would be magnified in CKD patients, especially those without residual kidney function. Those patients are deprived of clearing about 30 mcg aluminum per day, which are usually cleared into 2.5 L of urine by healthy Egyptians [34]. Eventually, these aluminum extra loads are being dumped daily into the bone and other organs without efficient clearance by HD. This would yield a tremendous cumulative aluminum dose, considering the multiplied effect of long dialysis vintage. This accordingly raises more questions about the potential extra-skeletal effects of aluminum on cardiovascular, neurological, hematological, endocrine, and other organs [35]. Unquestionably, we can say that aluminum bone disease in CKD is still present and has not become extinct yet, and we are not alone in that. The Brazilian Registry of Bone Biopsy (REBRABO) – where 29% of its cases were based on a research indication – showed significant aluminum accumulation in 38% of its cases [25, 36]. They also reported that aluminum accumulated in their patients as an independent predictor of cardiovascular events [35]. One of the rare reports for bone biopsy studies from Asia denies any aluminum accumulation in their biopsies. This was an unexpected result as 40% of their patients were maintained on aluminum-containing phosphate binders [14]. Accordingly, the optimistic notion of “extinct aluminum bone disease” cannot be generalized all over the whole globe. It should be kept in mind as a probable differential diagnosis.

**Fig. 6:**
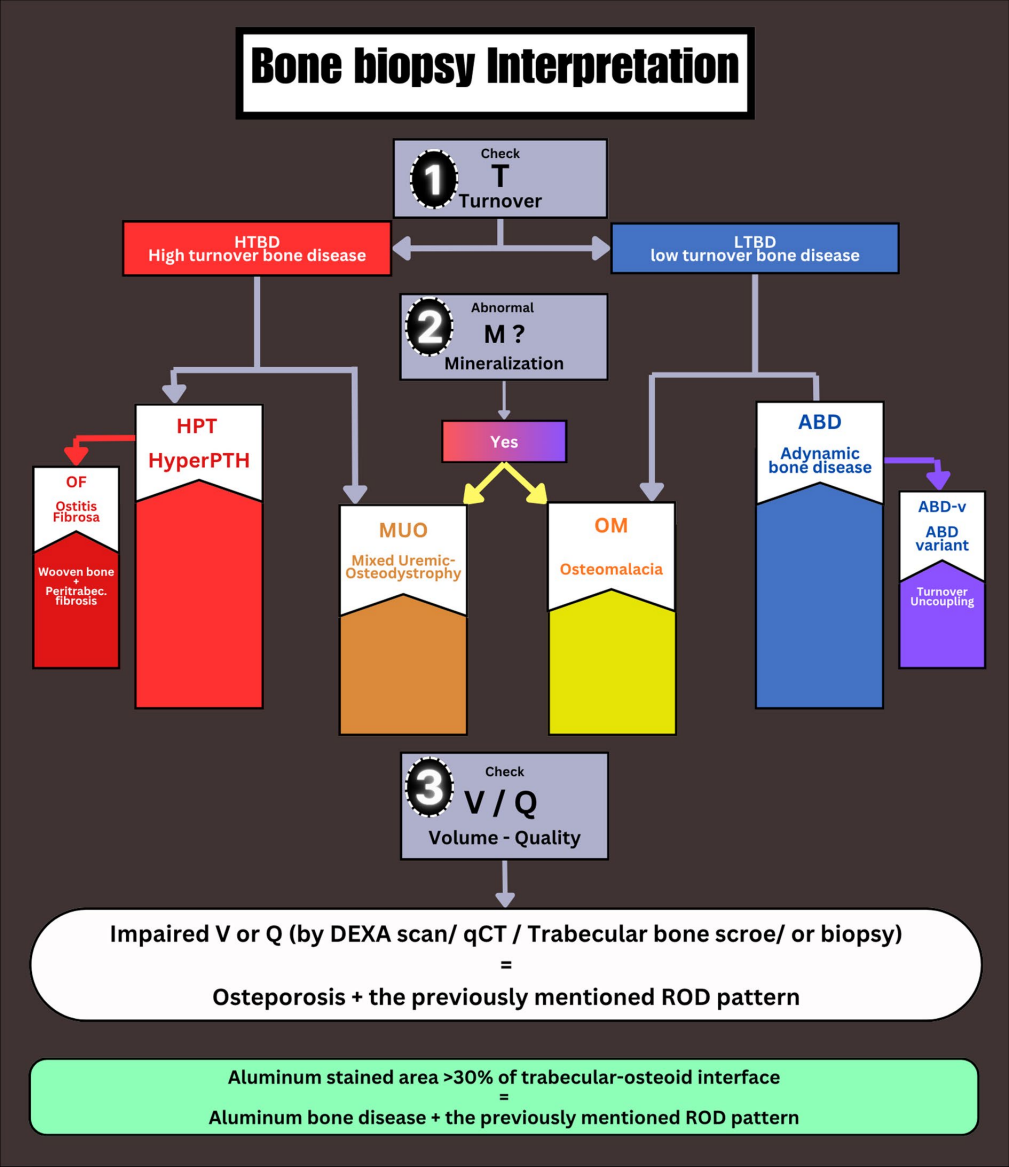
Bone biopsy interpretation and types of renal osteodystrophy. First, we check the T (turnover parameters). If high, we classify it as high-turnover bone disease (HTBD) and proceed to the left side of the algorithm. If mineralization is normal, we are dealing with hyperparathyroidism (HPT). Osteitis fibrosa (OF) is its ugliest face, with woven bone and peritrabecular fibrosis. If mineralization is defective in addition to the high turnover, we are dealing with mixed uremic osteodystrophy (MUO). On the right side of the algorithm, if turnover parameters are low, we have low-turnover bone disease. The main category here is adynamic bone disease (ABD), which is characterized by preserved mineralization. “Adynamic bone disease variant (ABD-V)” is a less-studied pattern that can be considered a more debilitating sector of adynamic bone disease. It is characterized by increased osteoclast activity and suppressed osteoblasts. This uncoupling carries a very high risk of bone loss. If mineralization is defective with low turnover, this represents Osteomalacia (OM). Finally, we check the parameters of reduced bone volume (V) or impaired quality to diagnose osteoporosis, which can also be speculated from other non-invasive imaging modalities such as DEXA scan

Aluminum accumulation was not the only similarity between our biopsied group and the Brazilian cohort. Usually, patients with ABD have low osteoblast and osteoclast actions. Although turnover is low in ABD, if there is symmetrical suppression of bone formation and resorption, bone volume might be preserved. Our patients had a unique turnover pattern. Most of our patients with ABD had high osteoclast and low osteoblast indices. High bone resorption and low bone formation rates may lead to negative bone balance and markedly reduced bone volume over time. A similar pattern was present in 40% of the Brazilian patients with ABD. This pattern was previously described as an adynamic bone disease-variant (ABD-V) [20]. A lot of questions need to be answered about the exact nature of this variant. Whether it is a unique disease pattern with a separate entity and outcome or just a transition phase from low to high turnover state, this may need further long-term prospective studies to answer. Whether this variant is a fact or a fiction, and whether aluminum has a role in its pathogenesis, reversal of this uncoupling is essential to stop the ongoing bone loss. Figure 6 summarizes our insight into the available classifications for ROD and the situation of these unique types (ABD-V, aluminum bone disease, OF) among the classic TMV classification.

### Study advantages

Although our biopsied cases are few, the study has multiple advantages. To the best of our knowledge, this is the first study that uses bone biopsy to investigate ROD not only in Egypt but also in Africa. Since international guidelines should adjust the management plan all over the world, the nature of ROD in these regions should be represented as well. These results should induce the launch of campaigns to reappraise the magnitude of aluminum toxicity in CKD in developing countries. One other advantage of this study is the root cause analysis, which uncovered the probable causes for this problem. This needs to be translated into actions by quality control units in drug manufacturing and in administrative entities that approve drugs for use by CKD patients. Governmental and non-governmental entities should revise the state of control of heavy metals loads after this report. Another advantage in this study is the analysis of the prescription pattern that is expected to be revised to fill these previously mentioned gaps. Furthermore, focusing on ABD-V, which was under-reported in the literature, might have a role in this cohort, which is another advantage for this study. As not all ABD are the same [37]. The current study not only undercover the issue of aluminum toxicity but also correlates it to novel turnover biomarkers and pathogenesis co-players. This led to the reporting of a significant association between FGF-23 and aluminum toxicity. Currently, we don’t have a mechanistic explanation for the high FGF-23 level in aluminum bone disease. Further experimental or large-scale clinical studies may help to explain this association and to generate potential hypothesis to explain this finding, which is limited by the small sample size. Moreover, this is the first study to report the CKD-MBD parameters in a group of PD patients in Egypt. Finally, besides the reported facts in this cohort, we included a simplified approach to interpreting bone biopsy reports that we expect to help general nephrologists and internists become familiar with different bone-related variables in Fig. 6. Besides a simplified review for the latest and most important clinically indicated biopsy-proven ROD cohorts all over the world, Fig. 5: ROD in the last 25 years.

### Limitations

This study faced several obstacles that limited the number of bone biopsies performed. The main barrier was patient refusal, largely due to the invasive nature of the procedure. In some cases, patients were dissuaded by second opinions from physicians who did not recognize the diagnostic value of bone biopsy. A larger biopsy cohort would have allowed for more robust correlations and broader geographic representation, and patients stratification. For example, sub-analysis of diabetic and non-diabetic patients, as they may have different sensitivity to PTH and other factors. Additionally, the study was subject to recall bias, as some variables—such as bone pain history and previous medication use—depended on patient memory. Determining the exact origin of pain and its severity was further complicated by subjective variations in pain thresholds. Another key limitation was the lack of quantitative histomorphometric data; only qualitative assessments from clinical reports were available. Comprehensive Quantitative Bone histomorphometric analysis is the best test to precisely describe the bone microarchitecture changes. Yet it is mainly a research tool rather than a clinical assessment tool. Due to its very high costs and lack of financial support for the current study, we couldn’t have a quantitative assessment for all parameters. Professors in the University of Kentucky had generously offered to do a qualitative assessment of the bone specimens for free as part of our collaboration with them within the ISN sistership program. This qualitative description was summarized in Fig. 2A. In the non-biopsied group, tests such as vitamin D levels, DEXA scans, and bone turnover markers were not uniformly performed due to resource constraints. Financial limitations restricted the study’s focus to the biopsied cohort. Finally, deferoxamine (DFO) testing, indicated by our findings, could not be implemented due to its unavailability in Egypt.

## Conclusion

Despite advances in CKD management, challenges in diagnosing and treating CKD-MBD remain, particularly in under-resourced settings. Bone biopsy, though underutilized, is essential for accurate diagnosis. This study confirms that aluminum bone disease persists and must be addressed through coordinated clinical, regulatory, and public health efforts. These results of high aluminum cannot be generalized to the whole spectrum of renal osteodystrophy in Egypt, as the invasive nature of the biopsy may lead to selection/indication bias. Those with mild or no bone pain or osteoporosis did not accept joining the biopsy group and were not offered the advanced biomarkers.

## Supplementary Information

Below is the link to the electronic supplementary material.

Supplementary Material 1

## Data Availability

The data supporting the findings of this study are available from the corresponding author upon reasonable request.

## Abbreviations

25-OH: Hydroxy (refers to 25-hydroxyvitamin D)
ABD: Adynamic Bone Disease
ABD-V: Adynamic bone disease variant
AL: Aluminum Negative group
AL+: Aluminum Positive group
BsAP: Bone -specific Alkaline Phosphatase
CKD: Chronic Kidney Disease
CKD-MBD: Chronic Kidney Disease - Mineral and Bone Disorder
DEXA: Dual-Energy X-ray Absorptiometry
ELISA: Enzyme-Linked Immunosorbent Assay
FGF23: Fibroblast Growth Factor 23
HD: Hemodialysis
HTBD: High Turnover Bone Disease
ICP: Inductively Coupled Plasma
iPTH: Intact Parathyroid Hormone
ISN: International Society of Nephrology
KDIGO: Kidney Disease: Improving Global Outcomes
LTBD: Low Turnover Bone Disease
MNDU: Mansoura Nephrology and Dialysis Unit
OM: Osteomalacia
PD: Peritoneal Dialysis
ROD: Renal Osteodystrophy
TRAP5b: Tartrate-Resistant Acid Phosphatase 5b
CT scan: Computerized tomography scan
